# Hepatic and Brain Spatial Gene Expression Changes in Intragastric Alcohol Fed APP/PS1 Alzheimer’s Disease Mouse Model

**DOI:** 10.21203/rs.3.rs-10760512/v1

**Published:** 2026-08-24

**Authors:** Ashley Duche, Devaraj Venkatapura Chandnra, Derick Han, Rachita K. Sumbria, Moom Rahman

**Affiliations:** Chapman University; Chapman University; Keck Graduate Institute; Chapman University; Chapman University

**Keywords:** Alzheimer’s Disease, Alcohol Drinking, Liver, Brain, Spatial Transcriptomics, APP/PS1 Mice, Intragastric Alcohol Feeding

## Abstract

**Background:**

Alcohol use is increasingly recognized as a significant modifier of Alzheimer's disease (AD) risk and progression. Two key organs, the liver and the brain, are central to understanding the impact of alcohol intake on AD. This is due to the liver being the primary site of alcohol detoxification and a major target of alcohol-induced injury, while the brain harbors the neuropathological hallmarks of AD. Although growing literature now links liver dysfunction to AD pathogenesis, the molecular mechanisms linking peripheral alcohol-induced liver injury to brain pathology remain poorly defined. To address this gap, we performed what is, to the best of our knowledge, the first integrated, multi-organ spatial transcriptomic analysis of liver and brain tissue from APP/PS1 AD mice subjected to chronic intragastric alcohol feeding.

**Methods:**

Following five-weeks of either control- or alcohol-diet feeding of APP/PS1 mice, differentially expressed genes (DEGs) were quantified in postmortem tissue across regions of interest (ROIs) spanning periportal and perivenous liver zones, along with Aβ plaque-bearing and Aβ plaque-free regions of the cortex and hippocampus in the brain. Pathway and network analyses were then used to identify candidate hub genes and biological processes altered within and across ROIs, followed by in silico nomination of therapeutic targets and drug repurposing compounds.

**Results:**

Following alcohol exposure, the most prominent transcriptional changes in the liver occurred in the perivenous zone, followed by the periportal zone. Among brain ROIs, the strongest differential expression occurred in the plaque-bearing hippocampus, with few or no DEGs detected in the remaining ROIs. These findings highlight Aβ pathology-dependent and region-selective tissue vulnerability to alcohol in the brain and liver during AD. Accordingly, cross-tissue comparisons focused on the plaque-bearing hippocampus and liver ROIs. This revealed coordinated molecular perturbations, including shared downregulation of *S100a8* and *Tmem267*, as well as opposing regulation of *Lrp1*, *Osgin1*, and *Cpsf7* between the plaque-bearing hippocampus and perivenous liver ROIs, respectively. Enrichment analyses indicated convergent dysregulation of cytoplasmic processes, metal ion homeostasis, redox/oxidative stress responses, mitochondrial pathways, and immune signaling. Although no gene-level overlap was observed, identified candidate therapeutic compounds and targets converged on pathways regulating metabolic sensing, kinase and phosphatase balance, proteostasis, inflammation, autophagy, and neurovascular signaling, which are central to aging biology, chronic alcohol exposure, and AD.

**Conclusion:**

These findings implicate significant liver–brain crosstalk through which chronic alcohol exposure may modulate AD-relevant pathology and reinforce the growing recognition of the liver as a critical organ in AD pathogenesis. Furthermore, these results reveal key alcohol-driven hepatic and brain gene perturbations and dysregulated pathways relevant to AD along with actionable therapeutic targets for future investigation.

## Introduction

Alzheimer’s disease (AD) is a progressive neurodegenerative disorder and the most common cause of dementia, affecting millions of people worldwide [1]. AD is characterized by the buildup of extracellular amyloid-beta (Aβ) plaques and formation of intraneuronal neurofibrillary tangles (NTFs) due to the hyperphosphorylation of tau proteins, leading to neuronal degeneration and loss [2]. While the amyloid hypothesis of AD posits that Aβ accumulation in the brain is the main driver of the disease, the primary molecular etiology of the disease remains unclear. The well-established contributions of both genetic and lifestyle risk factors further underscore the multifactorial and heterogeneous nature of AD [2].

Among the lifestyle factors, chronic alcohol consumption is a significant modifiable risk factor for AD [3, 4]. Moderate-to-heavy alcohol intake has been associated with increased brain Aβ load and NFTs in humans [5, 6]. Experimental studies corroborate these findings, and chronic alcohol consumption has been shown to modulate cerebral AD pathology directly by disrupting Aβ synthesis and clearance, and by inducing microglial and brain endothelial alterations [3]. For example, chronic alcohol feeding to APP23/PS45 double-transgenic AD mice elevated the expression levels of amyloid precursor protein (APP) and β-site amyloid precursor protein cleaving enzyme (BACE) in the brain [7]. Similarly, alcohol-fed APPswe/PSEN1dE9 (APP/PS1) mice showed an increase in brain atrophy, Aβ plaques, and N-methyl-D-aspartate receptor (NMDAR), with a decrease in γ-aminobutyric acid type-A receptor (GABAAR) mRNA levels [8]. This suggests that alcohol may increase Aβ deposition by disrupting brain metabolism and the balance between excitatory/inhibitory neurotransmission [8]. Furthermore, alcohol binges in rats decreased microglial count and caused microglial dystrophy [9]. Studies have also revealed that chronic alcohol consumption activated the TLR4/p38 MAPK pathway and impaired microglial phagocytosis of Aβ [10], and chronic alcohol intake in adult mice dampened microglial reactivity to tau [11]. Other mechanisms by which alcohol may contribute to AD pathology involve alterations in cAMP response element-binding protein pathway, activation of Akt/mTOR signaling, increased activity of glycogen synthase kinase-3β and cyclin-dependent kinase-5, and modulation of autophagy [3, 12]. Collectively, these findings underscore that chronic alcohol intake induces neurotoxic effects, thereby heightening susceptibility to AD-associated neuropathology.

Beyond its direct neurotoxic effects in AD, chronic alcohol exposure induces substantial metabolic dysfunction and liver injury, which are implicated in AD pathogenesis [13]. Accordingly, alcohol-induced liver injury, including inflammation, steatosis, fibrosis, or cirrhosis, disrupts key metabolic pathways, including glucose and lipid metabolism [14]. Such metabolic dysregulation has been associated with an elevated risk for AD, as evidenced by experimental findings demonstrating that chronic liver dysfunction in rats potentiates intraneuronal Aβ accumulation [15]. Similarly, plasma Aβ_40_ and Aβ_42_ levels were significantly increased in liver cirrhotic patients compared to healthy individuals, suggesting that hepatic dysfunction decreases peripheral Aβ clearance [16]. Consistent with this, chronic intragastric alcohol feeding to wild-type or double-transgenic APP/PS1 AD mice resulted in significant liver steatosis and decreased hepatic low-density lipoprotein receptor-related protein-1 (LRP1), the major route for peripheral Aβ clearance, with concomitant changes in brain AD biomarkers [17, 18]. Notably, hepatic LRP1 is shown to regulate brain Aβ levels [17, 19]. Beyond reducing hepatic LRP1, alcohol-induced liver injury can decrease serum albumin levels, which can increase circulating plasma Aβ [20, 21]. Furthermore, serum alanine aminotransferase levels (a marker of liver injury) in AD mice fed with the alcohol-Lieber-DeCarli diet were positively correlated with brain Aβ and neuroinflammation [22]. Collectively, these studies demonstrate that alcohol-induced hepatic dysfunction can directly drive AD pathology, underscoring the liver–brain axis as a relevant pathway in alcohol-driven AD.

**Figure 1:**
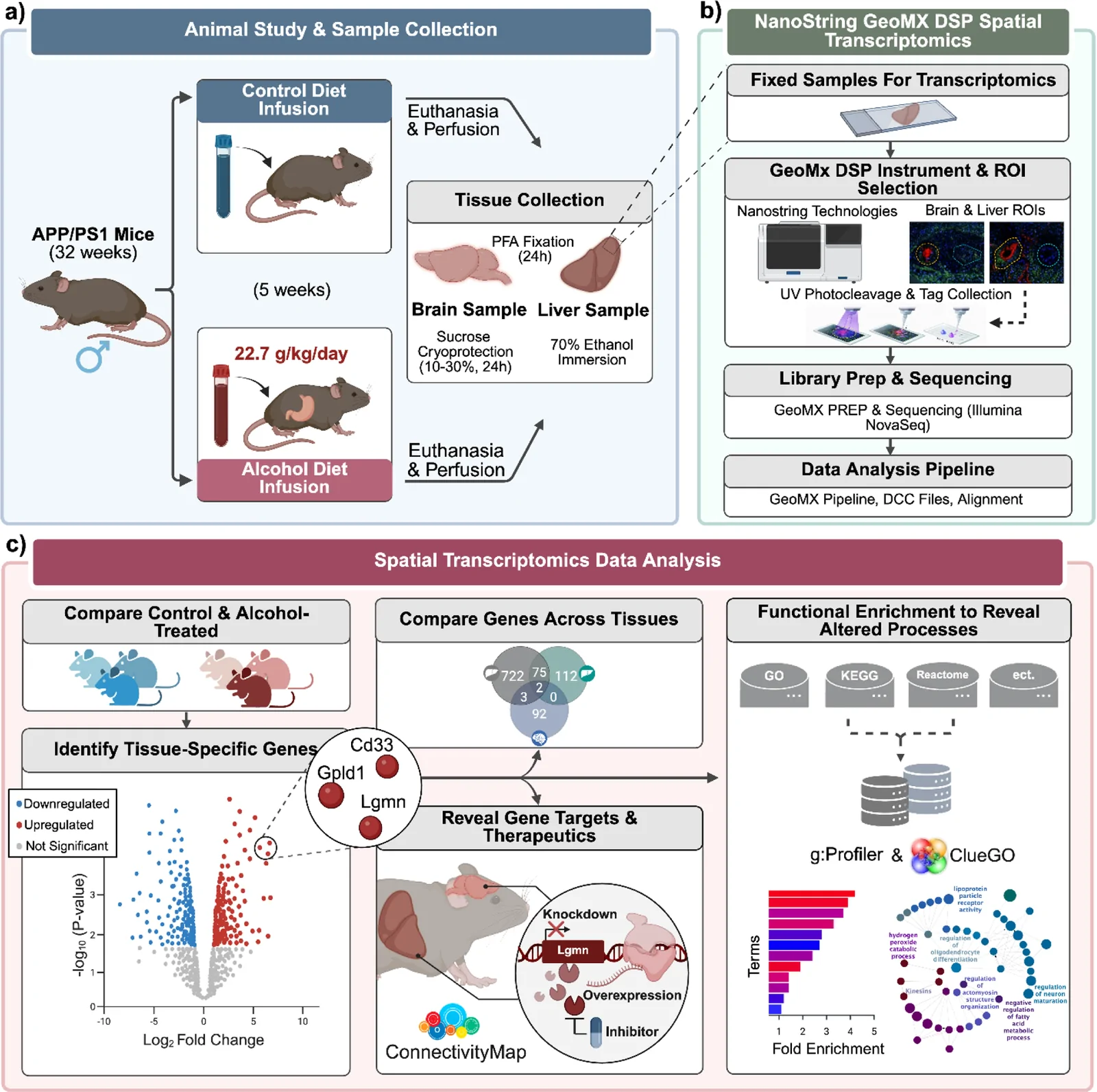
Study overview and analysis workflow. **a)** APP/PS1 mice were subjected to chronic alcohol administration, followed by brain and liver tissue collection. **b)** Collected tissues were processed using the NanoString GeoMx DSP, including library preparation, sequencing, alignment, and expression quantification. **c)** Generated datasets were analyzed using bioinformatics approaches to identify DEGs in brain and liver tissues. The identified DEGs were further characterized to reveal associated biological processes and pathways, and to highlight potential gene targets and therapeutic treatments. DSP: Digital Spatial Profiler; DEGs: Differentially Expressed Genes.

Therefore, although the majority of alcohol-related AD literature has focused on direct neurological mechanisms, alcohol-induced liver injury represents an important contributor to AD pathogenesis. However, the mechanisms by which alcohol-induced liver damage influences AD pathology remain poorly understood [23]. To address this gap, we performed what is, to the best of our knowledge, the first integrated, multi-organ spatial transcriptomic analysis of liver and brain tissue from AD mice subjected to chronic intragastric alcohol feeding. This is significant due to the liver’s role as the primary site of alcohol detoxification and a major target of alcohol-induced injury, while the brain is the principal site of AD neuropathological hallmarks. Thus, by applying spatial transcriptomics to both organs central to the interplay between chronic alcohol exposure and AD, we aimed to characterize coordinated transcriptomic changes and identify hepatic gene expression patterns that mirror or associate with region-specific AD brain pathology. For this, 32-week-old double-transgenic APP/PS1 AD male mice were fed alcohol intragastrically for five weeks, and postmortem liver and brain tissues were profiled for differentially expressed genes (DEGs) using the NanoString GeoMx^™^ Digital Spatial Profiler (DSP) platform (Fig. 1). Spatial transcriptomic changes were then mapped across plaque-bearing and plaque-free cortical and hippocampal brain regions, as well as key hepatic zones, the perivenous and periportal areas. Because alcohol-induced liver injury is spatially heterogeneous, with perivenous hepatocytes being more susceptible to injury than periportal hepatocytes [24], resolving molecular changes across both hepatic zones is essential. Together, integrated spatial transcriptomic profiling of region-specific liver and brain tissue provides a unique opportunity to elucidate how alcohol-induced liver dysfunction contributes to AD-relevant neuropathology through coordinated changes along the liver–brain axis.

## Materials and Methods

### Animal Study

Male double-transgenic (APPswe, PSEN1dE9; MMRRC Strain #034829-JAX) APP/PS1 mice, aged to 32 weeks, were used for this study [25]. Mice were purchased from the Jackson Laboratory (Bar Harbor, ME, USA) and housed in an animal room controlled for temperature and humidity, with *ad libitum* access to food and water. The experimental protocols were approved by the Southern California Research Center for Alcoholic Liver and Pancreatic Diseases and Cirrhosis Institutional Animal Care and Use Committee. Animals were randomized based on their age and body weight, housed individually, and categorized into two groups: the control group (received control diet) and the alcohol group (received alcohol diet) (n = 4). A sterile gastrotomy catheter was implanted in all animals for a constant long-term infusion of vehicle control or alcohol diet (22.7 g/kg/day), according to previously published methods [26, 27]. After five weeks of intragastric infusion of alcohol/vehicle control, the animals were euthanized (Euthasol, 150 mg/kg intraperitoneally) and perfused with cold phosphate-buffered saline (PBS), followed by collecting the brain and liver samples. The right hemibrains were fixed in 4% paraformaldehyde (PFA) for 24 h and cryoprotected using different concentrations of sucrose (10–30%) for 24 h each. Parts of the liver were fixed in 4% PFA for 24 h followed by immersion in 70% ethanol. All fixed samples were used for transcriptomics analysis.

### NanoString GeoMx Digital Spatial Profiler (DSP) Transcriptomic Analysis

The fixed frozen brain samples of control- and alcohol-treated APP/PS1 mice were sectioned to a thickness of 10μm and mounted onto the center of Super Frost Plus microslides (Fisher Scientific, 12–550-15, Waltham, MA, USA) with a 2–3mm distance between the sections, followed by overnight drying at room temperature. Similarly, to assess the various hepato-genomic changes of alcohol- or control-diet-fed APP/PS1 mice, the PFA-fixed liver tissues stored in 70% ethanol were embedded in paraffin and sectioned to a thickness of 5μm and mounted onto glass slides as described above. The slides were processed at the University of California, Irvine Genomics Research and Technology Hub according to the vendor’s specific protocols designed for the NanoString GeoMx DSP transcriptomics analysis. Briefly, the mounted brain sections were washed with PBS and alcohol, followed by antigen retrieval using 1x Tris EDTA (pH 9) and digested with proteinase-K to expose the RNA targets and later hybridized overnight with Mouse Whole Transcriptome Atlas probe set (catalog number 999064, NanoString Technologies, Inc., Seattle, WA, USA). Stringent washes were done to remove off-target probes with a mixture of formamide and 4x-saline sodium citrate, followed by staining with the morphology markers (Aβ and SYTO13 for nucleus). Then, the hybridized and stained slides were loaded onto the GeoMx-NGS DSP instrument (NanoString Technologies, Inc., Seattle, WA, USA) [28] and scanned to capture fluorescent images to select regions of interest (ROIs). For the brain, a total of 20 ROIs per experimental group with (plaque-bearing) or without (plaque-free) Aβ deposits were selected across the hippocampus and cortex. For the liver, a total of 16 ROIs per experimental group (8 perivenous and 8 periportal) were selected by scanning the processed liver sections on the DSP analyzer, followed by illumination for collecting the UV-photo cleavable oligonucleotide tags attached genomes. The collected ROI aspirates were processed as per the GeoMx-NGS readout library prep user manual (MAN-10117–05) and sequenced on the Illumina NovaSeq 6000 platform, targeting 200 reads per μm^2^ to get the spatially mapped digital counts (GSE324193). The FASTQ files thus generated were converted to Digital Count Conversion (DCC) files using GeoMx NGS pipeline software. Briefly, the reads were aligned to the GRCm38.p6 (Genome Reference Consortium Mouse Build 38; Patch 6) using the corresponding RTS-ID barcode list, and PCR duplications were removed and then converted to DCC files.

### Differential Gene Expression Analysis

To examine molecular alterations in brain and liver tissues, differential gene expression (DGE) analysis was then performed using DESeq2 (Version 1.42.1; R Version 4.3.3; RstudioVersion 4.3.3) [29–31]. For each ROI, control samples were compared with alcohol-treated samples to identify statistically significant DEGs. Multiple testing correction was applied using the Benjamini-Hochberg (BH) procedure to control the false discovery rate (FDR). Genes with an adjusted *p*-value (p-adj) ≤ 0.01 and an absolute log_2_ fold change (LFC) ≥ 0.32 were considered significant. However, tissue-specific DEG lists containing a limited number of genes (< 10) were excluded from downstream analyses. DEG lists with > = 10 genes were then compared across brain and liver ROIs to identify genes that were shared or unique to each. Finally, raw gene counts were normalized using DESeq2 and log_2_-transformed for heatmap visualization.

### Gene Co-expression Network and Hub Gene Analysis

Gene co-expression networks were generated using Cytoscape (Version 3.10.1) [32] with the GeneMANIA plugin (Version 3.5.3) [33]. This was done separately for each ROI using the identified DEGs, specifying *M. musculus* (mouse) as the species. Networks were visualized with nodes representing DEGs, color-coded by LFC, where deeper red indicates stronger upregulation and deeper blue indicates stronger downregulation. Node size also reflects the LFC, with larger nodes corresponding to higher absolute values.

Next, to identify genes central to the co-expression networks, CytoHubba [34] was used to calculate network topology measures. For each tissue-specific network, the top 10 genes were identified separately for degree, betweenness, and closeness centrality. These metrics reflect how connected a gene is, how often it is used to relay information across the network, and how close it is to other nodes, respectively. DEGs ranked in the top 10 across all three measures were considered hub genes. These genes are likely critical to network structure and may represent potential therapeutic targets due to their central roles.

### Connectivity Map (CMAP) Analysis

To explore potential therapeutic candidates, the web-based tool Connectivity Map (CMAP) [35] was used to compare our DEGs to gene expression signatures derived from small molecules and genetic perturbations generated in human cell lines. Due to the DEGs being identified from mouse tissue, the genes were first mapped to their corresponding human orthologs in R (Version 4.3.3) using Rstudio (Version 4.3.3) applying the orthogene package (Version 1.16.1) [36] to ensure compatibility with the reference database. Next, upregulated and downregulated genes from each tissue, mapped to their human orthologs, were queried against the CMAP database. CMAP returns connectivity scores (CS) that reflect similarity (CS ≥ 90) or dissimilarity (CS ≤ − 90) to our expression profiles. Thus, a CS ≥ 90 was used to identify significant gene overexpression profiles showing similarity to our captured disease signatures. In contrast, CS ≤ − 90 was used to identify significant compounds, drug classes, and gene knockdowns, suggesting the ability to reverse disease-related patterns captured in our tissue-specific signatures. This approach allows the identification of potential therapeutic gene targets and drug repurposing candidates.

### Functional Enrichment Analysis

To investigate the biological relevance of our DEGs, g:Profiler (Version e114_eg62_p19_27110d83) [37] was used to perform functional enrichment analysis. This web-based tool integrates multiple annotation sources, including Gene Ontology (GO), KEGG, Reactome, WikiPathways, CORUM, TRANSFAC, miRTarBase, and the Human Phenotype Ontology (HPO). Using g:SCS multiple testing correction method with a significance threshold of 0.05 and specifying *Mus musculus (mouse)* as the organism, statistically significant functional enrichment terms were identified for each ROI DEG list. These terms were then compared across tissues to identify shared or unique processes.

To resolve the net directionality of overlapping (bidirectional) enrichment terms identified in both up- and downregulated gene sets by g:Profiler, fast gene set enrichment analysis (FGSEA) was performed using fgsea (Version 1.38.0). The full DESeq2-ranked transcriptome was tested against *Mus musculus* gene sets (5–500 genes) retrieved using msigdbr (Version 26.1.0), including GO:BP, GO:CC, GO:MF, CP:KEGG_MEDICUS, CP:KEGG_LEGACY, CP:REACTOME, and CP:WIKIPATHWAYS. Because the fgsea gene-set collections do not correspond exactly to the g:Profiler annotation sources, the two analyses were treated as complementary rather than as a like-for-like replication.

Bidirectional g:Profiler terms were matched to the corresponding fgsea gene sets by term identifier. Identifier matching was reliable for GO terms, whose IDs are shared between g:Profiler and MSigDB; KEGG and Reactome identifiers differ between the two resources and were matched manually where possible and otherwise excluded from the automated cross-method comparison. Among matched terms, directionality was assigned as follows: bidirectional terms reaching a Benjamini–Hochberg-adjusted q < 0.05 in fgsea were classified as net up-regulated (NES > 1) or net down-regulated (NES < − 1) by the sign of the normalized enrichment score (NES); bidirectional terms that did not reach q < 0.05 in fgsea were classified as coordinated remodeling, consistent with opposing within-pathway regulation whose contributions cancel in the running-sum statistic.

To further expand on these findings, the Cytoscape plug-in ClueGO (Version 2.5.10) [38] was used to identify and cluster functionally enriched terms from the same DEG lists. This tool also integrates sources such as GO, KEGG, Reactome, WikiPathways, and CORUM, while allowing for the visualization of functionally grouped term networks. These networks are generated using kappa statistics, which groups terms based on shared genes and are used to represent functional similarity. These functionally related terms with shared genes are then clustered together and visualized using color-coded groupings to reflect their biological relatedness. Using this tool, enrichment was performed separately for each tissue, applying a significance threshold of p < 0.05 and a minimum Cohen's kappa coefficient ≥ 0.4 while correcting for multiple testing using the BH method. Lastly, enriched terms from each ROI were compared to identify overlapping and tissue-specific biological processes and pathways.

## Results

### Region-Specific Differential Gene Expression Analysis

**Figure 2:**
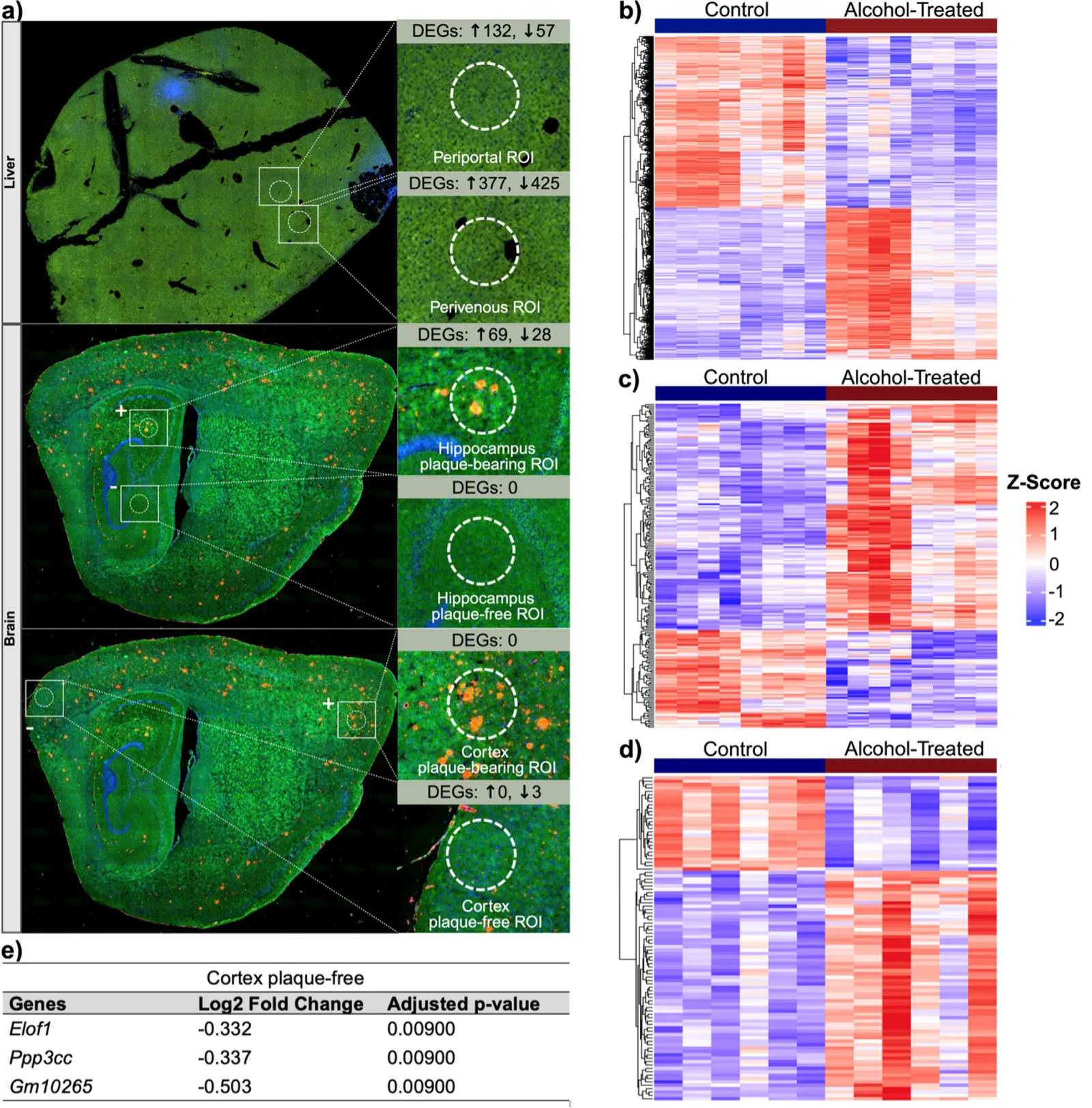
Tissue sections for each ROI with signature heatmaps. **a)** Representative liver section showing selection of periportal and perivenous ROIs, with the number of up- and down-regulated genes. This is followed by a representative sagittal brain section illustrating selection of plaque-bearing and plaque-free ROIs in the hippocampus and cortex, with a corresponding number of up- and down-regulated genes. The DEGs identified in each ROI define the corresponding gene signature, which is visualized as a heatmap for **b)** perivenous, **c)**periportal, and **d)** plaque-bearing hippocampus. Here, the top horizontal blue bar represents control samples, while the top horizontal red bar represents alcohol-treated samples respective to each ROI. Rows represent genes with upregulated expression shown in red, and downregulated expression in blue, with deeper colors indicating greater levels of upregulation or downregulation, respectively. **e)** Table highlighting DEGs identified for plaque-free cortex. DEGs: Differentially Expressed Genes; ROIs: Regions of Interest.

Overall, alcohol feeding induced substantially greater gene expression changes in the liver compared with the brain (Fig. 2a-d, Supplementary Table 1). Specifically, the perivenous ROI exhibited more than a 4-fold increase in DEGs (DEGs = 802; 377 upregulated and 425 downregulated) compared to the periportal ROI (DEGs = 189; 132 upregulated and 57 downregulated) in the alcohol-fed mice. Within brain regions, the plaque-bearing hippocampus ROI demonstrated the most robust transcriptional response with 97 DEGs (69 upregulated and 28 downregulated) between control and alcohol-treated mice. In contrast, the plaque-free cortex exhibited minimal gene expression changes, with only 3 downregulated DEGs identified (Fig. 2E). No statistically significant DEGs were detected in the plaque-bearing cortex or in plaque-free hippocampal regions. Hence, downstream pathway and network analyses were subsequently performed for the periportal, perivenous, and plaque-bearing hippocampal regions, which exhibited a sufficient number of DEGs to support robust analysis.

### Gene Co-expression Network and Hub Genes

Using the significant DEGs identified, gene co-expression networks were constructed for each ROI and used to identify critical hub genes within each network (Supplementary Fig. 1). The following hub genes were identified including *Cpsf7, Nbea, Stx1a, Nop56*, and *Oga*, for the plaque-bearing hippocampus, followed by *Cebpa, Cmbl, Cyp4v3, Gnmt, Mcee, Rnase4*, and *Suclg2* for periportal and *F5, Fn1, Hp*, and *Ubc* for perivenous (Table 1 and Supplementary Fig. 1).

These hub genes were identified through degree, betweenness, and closeness centrality measures. Genes appearing across all three measures were designated as hub genes, highlighting their central role in key processes within each network.

**Figure 3:**
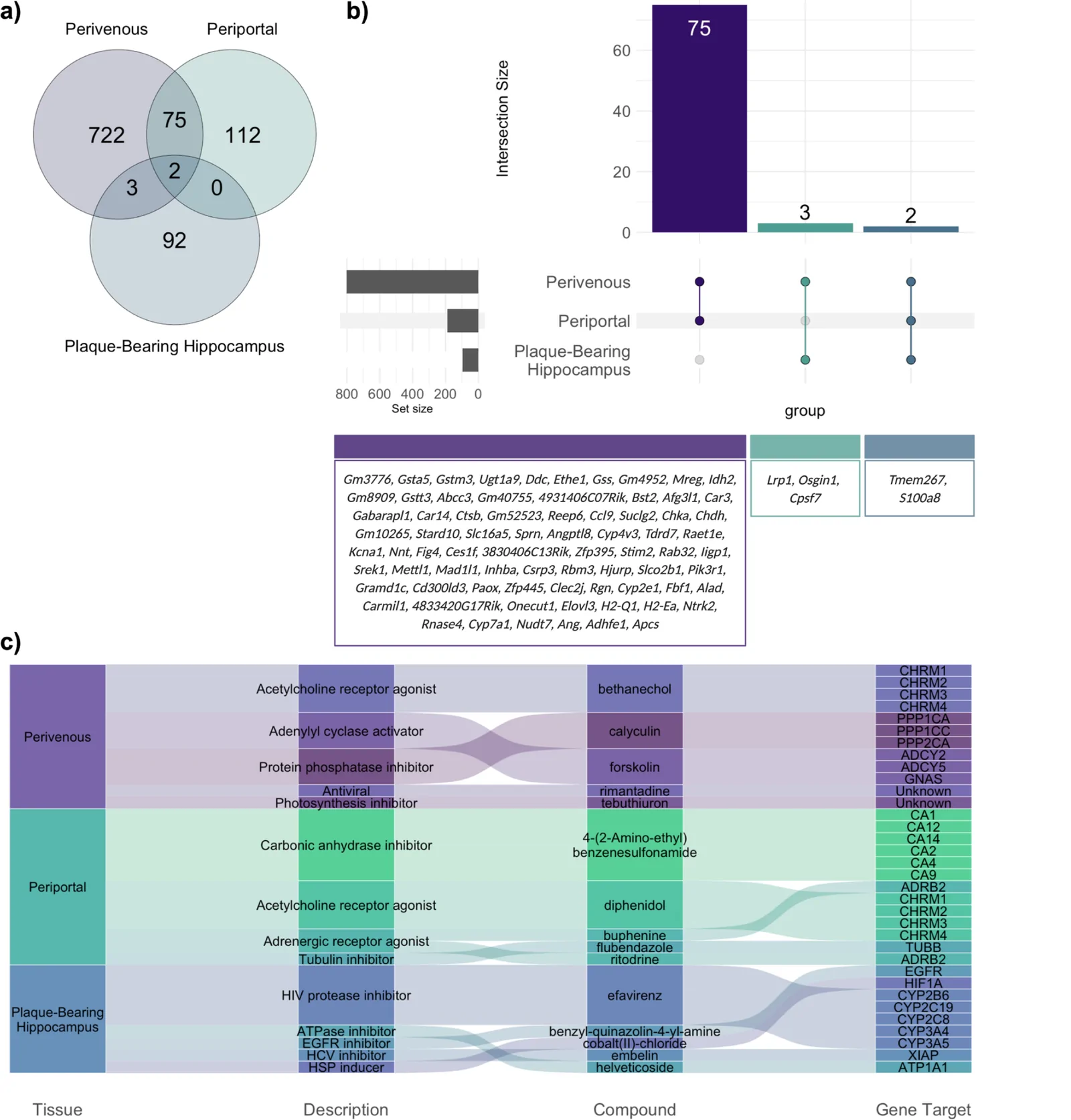
Gene overlaps across ROIs along with potential therapeutic candidates. **a)** Venn diagram showing the number of genes shared across brain and liver ROIs. **b)** UpSet plot displaying overlapping genes between ROIs where vertical bars represent the number of shared genes across specific tissue intersections, while horizontal bars indicate the total number of genes per ROI. Specific genes in each intersection are listed below the plot. **c)** CMAP analysis showing top 5 region-specific compounds across each ROI to potentially reverse disease-related gene expression captured in our signatures. ROI(s): Regions of Interest.

To examine shared molecular signatures across brain and liver tissues, overlapping DEGs were identified between the perivenous, periportal, and plaque-bearing hippocampus ROIs, as shown in Fig. 3a-b (Supplementary Table 2). This revealed two genes downregulated consistently across all tissues including *S100a8* and *Tmem267*. In addition, *Lrp1, Osgin1*, and *Cpsf7* were shared between the plaque-bearing hippocampus and perivenous tissues, showing opposing regulation across regions. This included upregulation of *Lrp1* and *Cpsf7*, while *Osgin1* was downregulated in the plaque-bearing hippocampus, whereas in the perivenous ROI, a downregulation pattern was observed. Together, these findings highlight distinct yet interconnected stress and repair mechanisms across brain-liver axis that may influence vulnerability to alcohol-related neurodegeneration. Finally, the largest overlap occurred between periportal and perivenous regions, including 75 additional genes. This overlap underscores the widespread alcohol-induced transcriptional alterations across liver ROIs, suggesting potential effects on multiple biological processes and organ systems beyond the brain.

### Potential Gene Targets and Therapeutics

CMAP analysis revealed compounds, gene knockdowns, and gene overexpression profiles that exhibited strong connectivity with disease-related transcriptional signatures across each ROI (Fig. 3c, Supplementary Table 3A-C). In the perivenous ROI, calyculin (protein phosphatase inhibitor), tebuthiuron (photosynthesis inhibitor), forskolin (adenylyl cyclase activator), rimantadine (antiviral), and bethanechol (acetylcholine receptor agonist) were strongly connected to disease-signature reversal (Supplementary Table 3A). For the periportal ROI, flubendazole (tubulin inhibitor) showed the strongest signature reversal (Supplementary Table 3B). Additional top hits clustered around receptor-mediated signaling, including ritodrine and buphenine (β2-adrenergic receptor agonists), diphenidol (muscarinic acetylcholine receptor modulator), and 4-(2-aminoethyl) benzenesulfonamide (carbonic anhydrase inhibitor). The plaque-bearing hippocampus revealed cobalt chloride (Heat Shock Protein inducer), efavirenz (HIV protease inhibitor), embelin (Hepatitis C Virus inhibitor), benzulquinazolin 4yl-amine (EGFR inhibitor) and helveticoside (ATPase inhibitor) as the strongest negative connectivity to our signature (Supplementary Table 3C). Next, for the perivenous ROI, gene knockdown signatures most strongly associated with reversal of our disease-related signature included *STAT5B*, *RPS13*, *GSR*, *SEC14L1*, and *SH3BP5* (Supplementary 3A) and then for periportal ROI included *SEC16A*, *TARBP1, BMI1, NR1D2*, and *ADAM10* (Supplementary Table 3B). Finally, the plaque-bearing hippocampus gene knockdown signatures included *E2F3*, *ABCA3*, *CAB39, ARPC1A*, and *MED1* (Supplementary Table 3C). In contrast, several gene overexpression profiles showed strong connectivity with the disease signatures, suggesting that targeting these genes may be therapeutically relevant. For the perivenous ROI, these included *PSMB10*, *HADHA, RALA, PHF17*, and *TCF7L2*. In the periportal ROI, *IFNB1*, *BCL10*, *HOXA9*, and *EHF* were identified. Finally, for the plaque-bearing hippocampus, the top overexpression profiles included *ETV1, STX4, UBAP1, HSD17B10*, and *DDB2* (Supplementary Table 3A-B).

### Shared Dysregulated Processes Across Tissues

**Figure 4:**
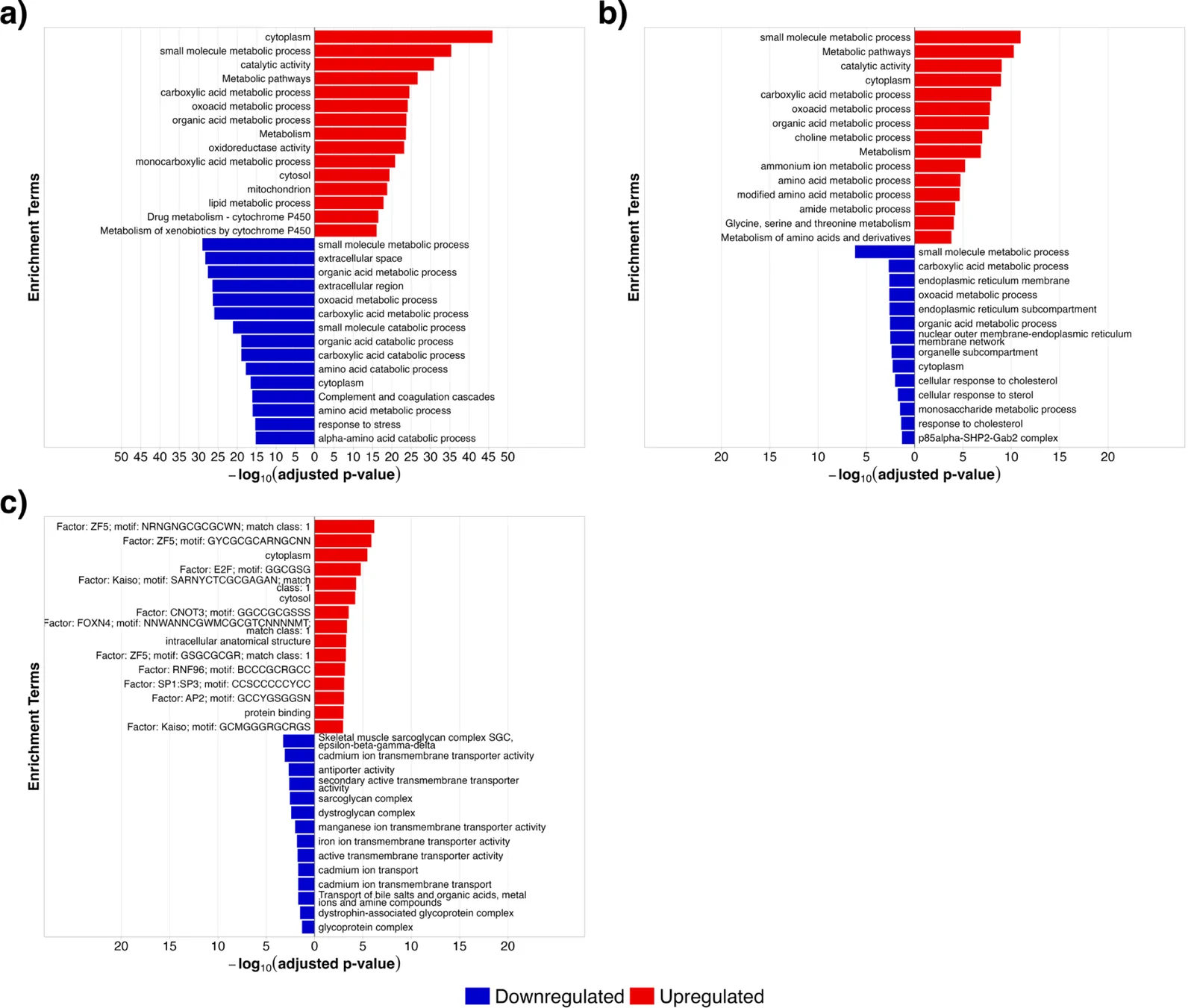
Top 15 enriched terms per ROI from g:Profiler overrepresentation analysis. Top 15 up- and down-regulated enrichment terms for **a)** perivenous, b) periportal, and **c)**plaque-bearing hippocampus ROIs. Each term was overrepresented among upregulated genes, downregulated genes, or both (bidirectional). Bidirectional terms were resolved to a net direction by GSEA, if available in the MSigDB collection (Supplementary Table 6). Bar length represents the –log_10_ g:SCS-adjusted *P* value for each term; up-regulated terms are shown in red and down-regulated terms in blue, with bidirectional terms appearing as both a red and a blue bar. ROI, region of interest.

Using the DEGs identified from each ROI, g:Profiler analysis was performed to characterize dysregulated processes and the direction of dysregulation, both shared and distinct across the perivenous, periportal liver tissues, and plaque-bearing hippocampus (Fig. 4a-c, Supplementary Table 4A-C and 6). This revealed shared upregulation of “cytoplasm”-related processes across all three ROIs, which also represented the sole overlap between the plaque-bearing hippocampus and periportal tissues. Notably, while this term was upregulated in the plaque-bearing hippocampus, in both liver zones it reached significance in the up- and down-regulated gene sets simultaneously (see Bidirectional Enrichment), and its net direction in the liver is therefore not unidirectional. In the perivenous and periportal comparison with the plaque-bearing hippocampus, additional overlap was observed: the plaque-bearing hippocampus and perivenous tissues shared enrichment of “protein binding”, “cytosol”, and “intracellular anatomical structure”. These were upregulated in the hippocampus; in the perivenous liver, “cytosol” and “intracellular anatomical structure” were likewise upregulated, whereas “protein binding” was bidirectional (significant in both directions) and is addressed with the other bidirectional terms below. Interestingly, the perivenous liver and plaque-bearing hippocampus also shared enrichment of transcription factor motifs, with coordinated upregulation of networks involving ZF5, Kaiso, E2F, FOXN4, SP1/SP3, WT1, BEN, and BCL6B. The overlap in transcription factor motifs between the plaque-bearing hippocampus and perivenous liver may indicate coordinated gene regulation in these regions, suggesting alcohol exposure may trigger parallel molecular responses in both neuronal and hepatic tissues.

**Figure 5:**
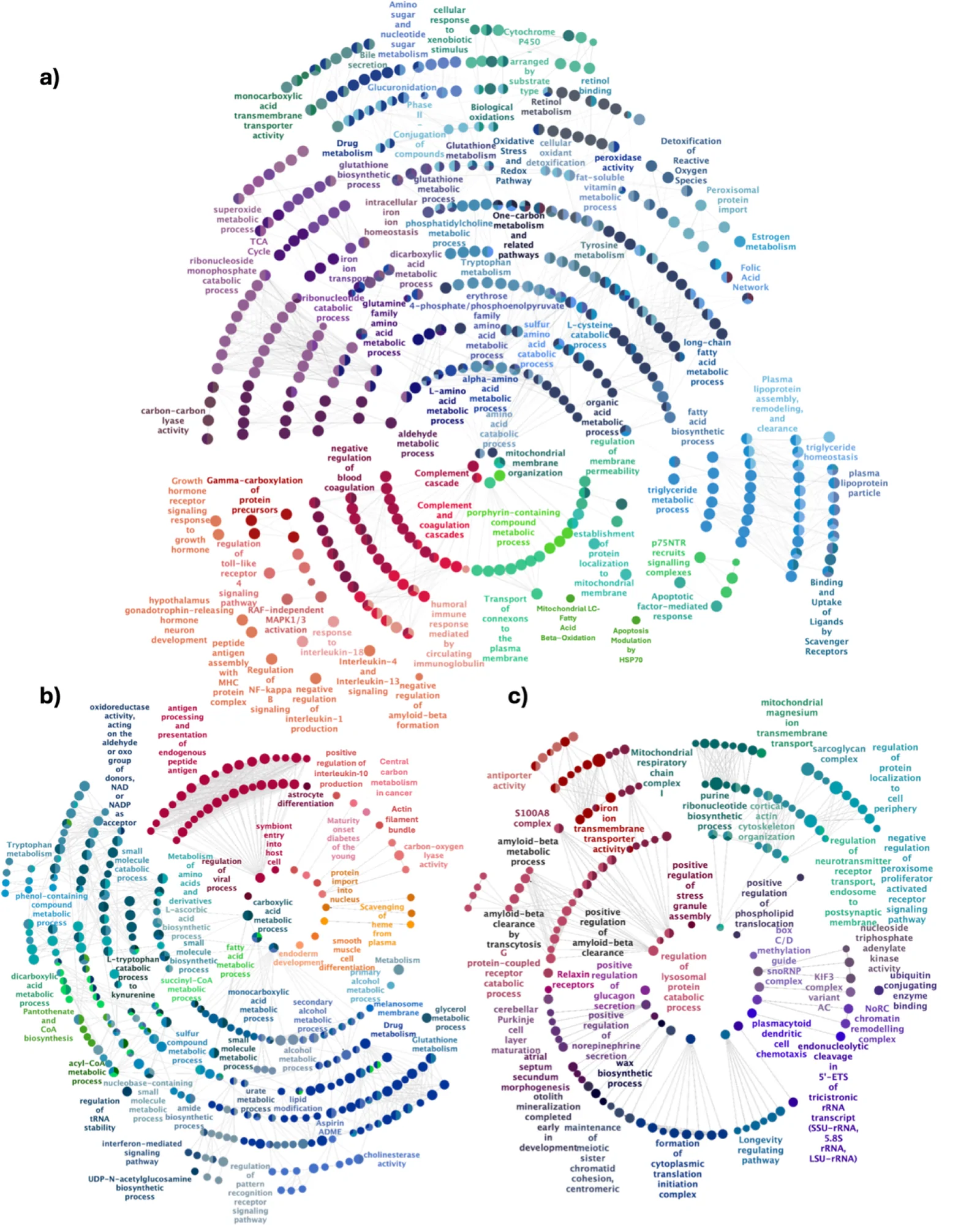
Functional network analysis of ClueGO enrichment terms for each ROI. Network analysis of enrichment terms highlighting biological processes overrepresented across tissues including **a)** perivenous, **b)** periportal, and **c)** plaque-bearing hippocampus ROIs. Each node represents an enriched biological process with edges connecting functionally related terms based on overlapping genes. Functional similarity is further reflected by shared color while node size reflects adjusted p-value, with larger nodes indicating stronger statistical significance. Only the top representative term is labeled for each group. ROIs: Regions of Interest.

Next, examining functional relationships of the dysregulated processes across tissues using ClueGO revealed shared dysregulation or adaptation of metal-related pathways, including “metal ion SLC transporters” and “metal sequestration by antimicrobial proteins” in perivenous and plaque-bearing hippocampus ROIs (Fig. 5a and 5c, Supplementary Table 5A and 5C). In perivenous, “metal ion SLC transporters” was linked to reduced expression of *Cp* (LFC = − 1.27), *Slc39a14* (LFC = − 0.73), and *Slc41a2* (LFC = − 0.75), with upregulation of *Slc39a2* (LFC = 0.93). In the plaque-bearing hippocampus, this term was associated with downregulation of *Slc11a1* (LFC = − 0.42) and *Slc11a2* (LFC = − 0.39). For “metal sequestration by antimicrobial proteins”, the perivenous region showed strong downregulation of *Lcn2* (LFC = − 3.43), *S100a8* (LFC = − 2.12), and *S100a9* (LFC = − 1.57), while the plaque-bearing hippocampus showed downregulation of *S100a8* (LFC = − 0.33). These findings may indicate a coordinated suppression of metal-handling pathways across hepatic and brain tissue aligning with the shared dysregulation of *S100a8* across all tissues.

Next, the perivenous and periportal liver ROIs shared 88 enrichment terms, including the amyloid-relevant process “negative regulation of amyloid-beta formation” (Fig. 5a-b, Supplementary Table 5A-B). In the perivenous region, this term was linked to the downregulation of *Igf1* (LFC = − 0.97) and *Ntrk2* (LFC = − 1.47), together with the upregulation of *Bin1* (LFC = −1.11). In contrast, the periportal region showed a consistent downregulation of *Ntrk2* (LFC = − 1.43) and upregulation of *Rtn4* (LFC = 0.33). Additionally, the plaque-bearing hippocampus ROI demonstrated enrichment of lysosomal protein catabolic regulation. This included amyloid-related processes such as “amyloid-beta metabolic process”, “positive regulation of amyloid-beta clearance”, and “amyloid-beta clearance by transcytosis” which was linked to upregulation of *Lrp1* and *Mgat3*.

### Network Analysis of Liver–Brain Communication Pathways

Interestingly, across brain and liver ROIs, a pattern of pathway enrichment was revealed involving redox and oxidative stress responses, mitochondrial metabolism, and immune signaling (Supplementary Table 5A-C). Although the specific enriched pathways differed by region, these functional categories were repeatedly observed. For example, in the perivenous region of the liver, redox and oxidative stress pathways were supported by terms such as “Oxidative stress and redox pathway”, “regulation of cellular response to oxidative stress”, “Glutathione metabolism”, and “Ferroptosis”. Mitochondrial metabolism was represented by pathways including “Mitochondrial LC-Fatty acid beta-oxidation” and the “tricarboxylic acid metabolic process”, along with “regulation of mitochondrial outer membrane permeabilization involved in apoptotic signaling pathway”. Immune signaling in this region was specific to innate immune pathways and included enrichment of “Regulation of complement cascade”, “regulation of toll-like receptor 4 signaling pathway”, and the “Terminal pathway of complement”.

Similarly, the periportal region showed enrichment of redox and mitochondrial processes, with oxidative stress-related terms including “Oxidative stress and redox pathway”, “Glutathione metabolism”, and “aldehyde dehydrogenase (NAD+) activity”. Mitochondrial-related pathways in this region included “Mitochondrial protein degradation”, “Mitochondrial fatty acid beta-oxidation”, and the “Citric acid cycle (TCA cycle)”. Immune signaling differed from the perivenous region and reflected a mixed innate and adaptive profile, which was supported by the “interferon-mediated signaling pathway”, “positive regulation of cytokine-mediated signaling pathway”, “regulation of toll-like receptor signaling pathway”, and “Antigen processing and presentation”. Taken together, these findings suggest that alcohol exposure disrupts a common set of processes across tissues, redox and oxidative stress, mitochondrial-related processes, and immune signaling, while also differentially affecting each region.

Finally, in the plaque-bearing hippocampus, enrichment of oxidative stress pathways was evident and included terms such as “Oxidative stress induced senescence”, “RHO GTPases activate NADPH oxidases”, “KEAP1–NFE2L2 pathway”, and “Metal ion SLC transporters”. Mitochondrial energy metabolism was also represented by pathways such as “mitochondrial electron transport, NADH to ubiquinone”, “proton motive force-driven mitochondrial ATP synthesis”, and “Oxidative phosphorylation”. Immune-related enrichment in this region primarily reflected innate and inflammatory processes, including “positive regulation of dendritic cell antigen processing and presentation”, “astrocyte activation involved in immune response”, and “neutrophil aggregation”.

A subset of enrichment terms reached significance for both the up- and down-regulated gene sets within a region (bidirectional terms). These were confined to the liver and concentrated in the perivenous zone (86 of 91 terms; 5 periportal), and were predominantly GO processes spanning hepatic metabolism, transport, and redox-related functions (Supplementary Table 6). Because a term can be driven by different genes in each direction, we used signed GSEA to resolve net directionality where the term was represented in the MSigDB gene-set collection. Of the 91 bidirectional terms, 24 (26%) were evaluable by GSEA while the remaining 67 lacked a corresponding MSigDB gene set and could not be adjudicated. Among the evaluable terms, 11 resolved to a net direction (q < 0.05; |NES| 1.40–1.86): net induction of lipid and metabolic processes including fatty acid metabolic and biosynthetic processes, sulfur-compound and purine-compound metabolism, and response to xenobiotic stimulus, alongside net suppression of organic-acid and small-molecule catabolism and response to nutrient levels. The remaining 13 evaluable terms did not reach significance in GSEA (q = 0.07–0.45) despite passing the bidirectional criterion, with up- and down-regulated member genes near-evenly balanced (fraction up-regulated 0.42–0.60). These included lipid catabolic process, steroid metabolic process, organic-acid biosynthesis, and iron-ion binding, all of which were classified as coordinated remodeling, due to opposing within-pathway regulation that cancels in the GSEA running-sum statistic. Together, these results indicate that within the perivenous hepatocyte compartment, alcohol exposure drives both net-directional shifts in lipid and xenobiotic metabolism and coordinated bidirectional remodeling of catabolic and redox-associated processes.

## Discussion

Results from this study indicate that chronic alcohol exposure drives region-specific transcriptional alterations in APP/PS1 mice, manifesting distinct intra-organ heterogeneity across both the brain and liver. The most pronounced transcriptional dysregulation was concentrated in the hepatic zones and the Aβ plaque-bearing hippocampus. Within the brain, the relative lack of transcriptional changes in the cortex contrasts with the significant changes observed in the hippocampus. While the hippocampus is particularly vulnerable to AD pathology, our findings reveal that alcohol exposure specifically exacerbates transcriptional changes in Aβ plaque-laden hippocampal regions [39]. In the liver, heightened transcriptional changes in the perivenous compared with periportal liver regions are consistent with the established susceptibility of perivenous hepatocytes to alcohol-induced injury [40, 41]. Together, these findings highlight Aβ pathology-dependent and region-selective vulnerability of the brain and liver to chronic alcohol exposure in AD.

Interestingly, across these three ROIs (plaque-bearing hippocampus, and perivenous and periportal liver zones), *S100a8* and *Tmem267* were consistently downregulated, suggesting potential suppression of immune- and inflammatory-related signaling pathways. S100 proteins regulate various processes such as APP processing, Aβ aggregation, tau phosphorylation, cytokine signaling, metal homeostasis, and innate immunity [42–44]. While *S100a8* is often upregulated in AD, where it contributes to Aβ aggregation and neuroinflammation [43], its downregulation here may reflect changes driven by chronic alcohol exposure and indicate dampened myeloid activation [45]. Prior studies show that S100A8/A9 can amplify myeloid responses through receptors such as TLR4 and RAGE [46, 47], promoting microglial phagocytosis [48]. Besides, *in vitro*, S100A8 and S100A9 can bind Aβ and suppress its fibrillization, potentially impacting Aβ accumulation [49]. The reduced *S100a8* expression in alcohol-fed mice in our study may alter Aβ phagocytosis and clearance, as well as Aβ aggregation dynamics, aligning with the increase in Aβ load observed in intragastrically alcohol-fed APP/PS1 AD mice [17].

While *Tmem267* gene remains poorly characterized in AD, prior research shows that *Tmem267* can induce the expression of *Aldh1al*, which encodes the enzyme ALDH1A1 involved in acetaldehyde metabolism and alcohol detoxification [50, 51]. In the brain, *Aldh1a1* reduction is associated with neurotoxicity, and ALDH1A1 activity is decreased with increased AD severity [52–54]. Further, epigenetic studies identified a large differentially methylated region within *Tmem267* in the hippocampus, where increased methylation correlated with reduced *Tmem267* expression and decreased microglial gene expression (*Hexb*, *Cd180*), suggesting a role in neuroinflammatory regulation [54]. Interestingly, microglial depletion with PLX5622 was shown to induce hepatic *Aldh1a1* in alcohol-fed C57 mice. Collectively, a reduction in *Tmem297* may exacerbate alcohol-induced hepatic injury and neurotoxicity by downregulating *Aldh1a1* and microglial genes, and may be an important link between alcohol intake and AD.

Notably, shared dysregulation in *Lrp1*, *Cpsf7*, and *Osgin1* was also observed between the plaque-bearing hippocampus and perivenous ROIs, but with opposing directions of expression, highlighting tissue-specific regulatory differences in response to chronic alcohol consumption. *Lrp1* encodes the well-characterized endocytic receptor, LRP1, that plays a key role in Aβ clearance, both at the blood-brain barrier (BBB) and peripherally in the liver [17, 55, 56]. Therefore, the observed downregulation of hepatic *Lrp1* with chronic alcohol exposure can impair peripheral Aβ clearance and promote its accumulation in the periphery and brain [17, 56]. In contrast, the observed upregulation of *Lrp1* in the hippocampus herein may reflect a localized response to increased Aβ load found in neural tissue following intragastric alcohol feeding to APP/PS1 mice [17, 56]. This aligns with previous studies showing that LRP1 regulation was site- and cell-type specific [57], including upregulation in astrocytes and vascular cells near Aβ plaques, potentially to enhance local Aβ clearance, with concurrent downregulation in neurons [57, 58].

In contrast to the established role of *Lrp1* in AD, little is known about the role of *Cpsf7* in AD pathology. *Cpsf* is known to encode a subunit of the cleavage and polyadenylation complex, which is essential for mRNA processing. In our analysis, its opposing regulation in the liver and brain following alcohol intake may reflect broader transcriptional adaptation to stress and needs further investigation. Finally, *Osgin1*, a p53-regulated gene involved in oxidative stress response, is upregulated in chronic liver disease, which aligns with our study [59]. Although the role of *Osgin1* in AD remains largely unexplored, one study reported its protective effect in human astrocytes under central nervous system (CNS) oxidative stress [60], suggesting it may contribute to neuroprotective mechanisms, which may be downregulated with chronic alcohol intake in the current study.

Next, CMAP analysis based on orthologous genes from APP/PS1 mice identified potential therapeutic compounds and intervention targets across brain and liver tissues relevant to alcohol intake. These results are best used for hypothesis generation rather than evidence of causal mechanisms. In the perivenous ROI, compounds were pharmacologically diverse and less centered on a single mechanism. Among the compounds returned for this region, forskolin, an adenylyl cyclase activator, targets cAMP signaling, a pathway associated with alcohol-related injury and associated neuropathology [61, 62]. Prior studies have also reported an improvement in behavioral and neuropathological outcomes in APP/PS1 mice with forskolin, although direct evidence linking hepatic forskolin responses to improved brain outcomes is still lacking [61, 62]. In comparison, compounds returned from the periportal ROI revealed a common pattern centering around receptor-mediated or signaling-related processes highlighted by β2-adrenergic receptor agonists (ritodrine and buphenine), and muscarinic acetylcholine receptor modulator (diphenidol). These compounds target pathways regulating hepatic lipid metabolism and inflammatory stress responses, both of which are disrupted in alcohol-associated liver disease [63–65]. In turn, this can lead to an increase in circulating peptides, cytokines, and pro-inflammatory signals promoting neuroinflammation and intensifying Aβ- and tau-related pathology in the brain [64–66]. Finally, for the plaque-bearing hippocampus, a diverse set of compounds were returned which highlighted efavirenz and embelin, both of which have been studied in AD. Efavirenz, a HIV protease inhibitor, has been studied as a CYP46A1 activator to enhance brain cholesterol turnover in early AD [67], while embelin, a HCV inhibitor, has shown neuroprotective and anti-Aβ effects in experimental AD models [67]. Notably, these compounds have not been reported in the context of alcohol-dependent AD. Next, across the top CMAP gene perturbation results returned for each region, no gene was shared across tissues, suggesting that the dominant trend is potentially region-specific rather than a shared liver-brain mechanism. However, across all compounds, there is strong convergence on pathways regulating cellular stress responses, metabolic sensing, kinase signaling, inflammation, autophagy, and neurovascular/immune modulation, which are central to aging-related liver-brain dysfunction, alcohol toxicity, and AD pathology. Overall, these findings highlight potential therapeutic targets and strategies open to further investigation to determine their relevance to alcohol-dependent AD.

Finally, dysregulated processes across tissues were revealed from g:Profiler. This included consistent upregulation of terms related to cytoplasmic activity across all tissues, suggesting heightened intracellular activity. However, a broader overlap in dysregulation was observed between the plaque-bearing hippocampus and perivenous regions including “protein binding,” “cytosol,” and “intracellular anatomical structure,” along with transcription factor motifs like SP1/SP3 and E2F. These transcription factors are regulators of genes involved in cell cycle control, oxidative stress, and neurodegenerative processes, including those implicated in AD [68, 69]. Chronic alcohol exposure has also been shown to alter SP1/SP3 and E2F activity, potentially compounding their impact on neurodegenerative pathways in AD [70].

In addition to the broader overlap of intracellular terms and shared TF motifs (SP1/SP3, E2F), ClueGO analysis revealed convergent dysregulation of metal homeostasis pathways in both perivenous liver and plaque-bearing hippocampus regions. Region-specific changes in solute carrier (SLC) family transporters (*Slc39a14, Slc41a2, Slc11a1, Slc11a2*) alongside coordinated suppression of metal-sequestering antimicrobial proteins (*S100a8, S100a9, Lcn2*) suggest disrupted divalent metal handling and compromised innate immune defenses across both tissues [71–73]. These findings are particularly relevant given the established role of *Lcn2* in liver–brain communication, where it can trigger HMGB1-TLR4-Nox2-mediated oxidative stress in neural tissue [72, 74]. The consistent downregulation of *S100a8* across both organs is especially notable, as it may reflect systemic metal dyshomeostasis that simultaneously impairs microglial Aβ clearance while enhancing metal-catalyzed neurotoxicity [72]. Together, these support metal imbalance and immune dysregulation as shared features of hepatic and neural responses in our mouse model, warranting further exploration in chronic alcohol intake and AD.

Furthermore, recurrent dysregulation of redox/oxidative stress-related pathways, altered mitochondrial respiration and energy metabolism, and immune signaling were observed from our ClueGO network analysis. Specifically, for the perivenous liver region, enrichment terms characterized ferroptosis, glutathione metabolism, altered redox reaction, mitochondrial fatty acid β-oxidation/TCA cycle, and innate immune activation through complement and interleukin pathways. Similarly, the periportal liver region enrichment terms were associated with redox/oxidative stress response, glutathione metabolism, and mitochondrial metabolism (β-oxidation/TCA) but focused more on innate-adaptive immune processes, including antigen presentation and T cell–mediated responses. In the plaque-bearing hippocampus, dysregulated processes pointed toward oxidative stress and neuroimmune activation from altered divalent metal handling, mitochondrial oxidative phosphorylation and complex I assembly, accompanied by glial and immune involvement through astrocyte activation, and neutrophil aggregation. In prior studies, alcohol-induced hepatic inflammation and oxidative stress have been associated with BBB disruption, which may increase CNS exposure to circulating peripheral inflammatory mediators and promote neuroimmune activation [75, 76]. Separately, liver iron/redox pathway enrichment (e.g., ferroptosis and glutathione) and brain metal-handling signatures may reflect altered iron/redox biology across tissues, a process commonly associated with oxidative stress, mitochondrial dysfunction, and inflammatory signaling [76–78]. Collectively, these network-level alterations reflect parallel liver and brain changes under chronic alcohol exposure in AD, including hepatic metabolic and immune signatures alongside AD-relevant neurodegenerative pathways, and further analyses are needed to determine shared molecular mechanisms.

Nevertheless, this study has several limitations. First, transcriptomic profiling captures mRNA expression changes that may not fully reflect protein-level alterations. The dataset was derived from a small cohort of male transgenic mice, and future studies in larger cohorts of male and female mice will increase statistical power and account for sex differences. The APP/PS1 mice used herein overexpress human APP at non-physiological levels and do not capture tau pathology at the age studied. Next, drug repurposing predictions were based on gene expression signature similarity using the CMAP human cell lines and were not functionally validated. Furthermore, interpretations involving gene knockdowns, overexpressions, and therapeutic relevance need to be validated in follow-up studies. Directional resolution of the bidirectional terms is constrained by MSigDB coverage (only 24 of 91 terms were GSEA-evaluable) and the coordinated-remodeling label remains inferential, since a null GSEA result cannot fully distinguish balanced bidirectional regulation from weak signal. Notably, as this study represents the first liver-brain transcriptomic comparison in alcohol-fed AD mice, the DEGs identified across tissues were not externally validated using independent datasets, given that comparable data were not available at the time of analysis. Finally, tissues were included in downstream analyses only when they yielded a sufficient number of DEGs, potentially excluding biologically relevant but underpowered regions and pathways.

## Conclusion

This study used spatial transcriptomic profiling to characterize gene expression changes across the liver and brain of AD mice subjected to chronic intragastric alcohol administration. To our knowledge, this is the first integrated, multi-organ spatial transcriptomic analysis of alcohol-related AD pathology. This multi-organ approach enabled examination of liver expression patterns potentially reflecting AD-associated brain changes, and was motivated by the growing literature showing the role of liver dysfunction in AD. We identified differentially regulated genes across periportal and perivenous liver regions, and plaque-bearing hippocampus and plaque-free cortex. In the liver, altered expression was more pronounced in the perivenous than periportal region, consistent with known alcohol metabolism patterns. In the brain, dysregulated genes were largely confined to the plaque-bearing hippocampus, underscoring its heightened vulnerability to alcohol-dependent AD pathology. Cross-organ analysis revealed coordinated molecular disruptions, including consistent downregulation of *S100a8* and *Tmem267*, and opposing regulation of *Lrp1*, *Osgin1*, and *Cpsf7* between the plaque-bearing hippocampus and perivenous liver regions. Convergent dysregulation was observed across cytoplasmic processes, metal ion homeostasis, redox/oxidative stress responses, mitochondrial pathways, and immune signaling. Several potential therapeutic compounds and gene targets were subsequently identified. Collectively, these findings highlight relevant pathways and targets within the liver–brain axis through which alcohol may modulate AD-relevant pathology.

## Supplementary Material

Supplementary Files

This is a list of supplementary files associated with this preprint. Click to download.

• 5426GraphicalAbstract.tiff

• AdditionalFile1.docx

## Figures and Tables

**Table 1:** Tissue-specific hub genes.

Tissues	Hub Genes
Perivenous	*F5, Fn1, Hp, Ubc*
Periportal	*Cebpa, Cmbl, Cyp4v3, Gnmt, Mcee, Rnase4, Suclg2*
Plaque-Bearing Hippocampus	*Cpsf7, Nbea, Stx1a, Nop56, Oga*

## Data Availability

All data supporting the findings of this study, including raw FASTQ files and processed feature counts, are publicly available through NCBI GEO under accession number GSE324193.

## Abbreviations

AD: Alzheimer’s Disease
DEGs: Differentially Expressed Genes
ROI(s): Regions of Interest
CMAP: ConnectivityMap
Aβ: Amyloid-Beta
NTFs: Neurofibrillary Tangles
APP: Amyloid Precursor Protein
BACE: β-Site Amyloid Precursor Protein Cleaving Enzyme
APP/PS1: APPswe/PSEN1dE9
NMDAR: N-methyl-D-aspartate Receptor
GABAAR: γ-aminobutyric Acid Type-A Receptor
LRP1: Low-Density Lipoprotein Receptor-Related Protein-1
DSP: Digital Spatial Profiler
PFA: Paraformaldehyde
DCC: Digital Count Conversion
GRCm38.p6: Genome Reference Consortium Mouse Build 38; Patch 6
DGE: Differential Gene Expression
BH: Benjamini-Hochberg
FDR: False Discovery Rate
P-adj: Adjusted P-Value
LFC: Log_2_ Fold Change
CS: Connectivity Scores
GSEA: *Gene Set Enrichment Analysis*
GO: Gene Ontology
HPO: Human Phenotype Ontology
SLC: Solute Carrier
TCA: Citric Acid Cycle
BBB: Blood Brain Barrier
CNS: Central Nervous System
